# Tramadolium picrate

**DOI:** 10.1107/S1600536812004138

**Published:** 2012-02-04

**Authors:** B. P. Siddaraju, Grzegorz Dutkiewicz, H. S. Yathirajan, Maciej Kubicki

**Affiliations:** a Department of Studies in Chemistry, University of Mysore, Mysore 570 006, India; b Department of Chemistry, Adam Mickiewicz University, Grunwaldzka 6, 60-780 Poznań, Poland

## Abstract

In the title salt {systematic name: [2-hy­droxy-3-(3-meth­oxy­phen­yl)cyclo­hexyl­meth­yl]dimethyl­aza­nium 2,4,6-trinitro­phenol­ate}, C_16_H_26_NO_2_^+^·C_6_H_2_N_3_O_7_^−^, the cation is protonated at the N atom. The cyclo­hexane ring adopts a chair conformation with the hy­droxy substituent in an axial position. In the crystal, O—H⋯O and N—H⋯O hydrogen bonds link the cations and anions into supra­molecular chains along [100].

## Related literature

For general background to tramadol, see: Scott & Perry (2000 ▶). For related tramadolium crystal structures, see: Bica *et al.* (2010 ▶); Siddaraju *et al.* (2011 ▶). For asymmetry parameters, see: Duax & Norton (1975 ▶). For the Cambridge Structural Database, see: Allen (2002 ▶).
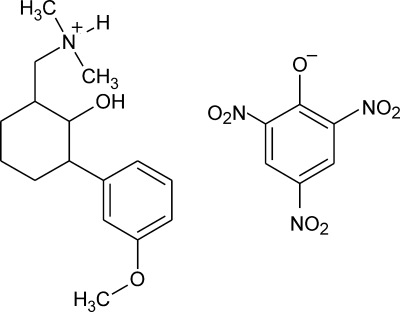


## Experimental

### 

#### Crystal data

C_16_H_26_NO_2_^+^·C_6_H_2_N_3_O_7_^−^
*M* *_r_* = 492.48
Triclinic, 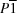

*a* = 8.5674 (10) Å
*b* = 12.3664 (12) Å
*c* = 13.2276 (13) Å
α = 113.003 (9)°
β = 107.686 (10)°
γ = 95.541 (9)°
*V* = 1191.4 (2) Å^3^
*Z* = 2
Mo *K*α radiation
μ = 0.11 mm^−1^
*T* = 295 K
0.35 × 0.2 × 0.15 mm

#### Data collection

Agilent Xcalibur Eos diffractometer
Absorption correction: multi-scan (*CrysAlis PRO*; Agilent, 2011 ▶) *T*_min_ = 0.974, *T*_max_ = 1.000
8566 measured reflections
4838 independent reflections
3621 reflections with *I* > 2s(*I*)
*R*_int_ = 0.011

#### Refinement

*R*[*F* ^2^ > 2σ(*F* ^2^)] = 0.052
*wR*(*F* ^2^) = 0.151
*S* = 1.02
4838 reflections
417 parameters
H atoms treated by a mixture of independent and constrained refinement
Δρ_max_ = 0.37 e Å^−3^
Δρ_min_ = −0.27 e Å^−3^

### 

Data collection: *CrysAlis PRO* (Agilent, 2011 ▶); cell refinement: *CrysAlis PRO*; data reduction: *CrysAlis PRO*; program(s) used to solve structure: *SIR92* (Altomare *et al.*, 1993 ▶); program(s) used to refine structure: *SHELXL97* (Sheldrick, 2008 ▶); molecular graphics: *XP* (Sheldrick, 2008 ▶); software used to prepare material for publication: *SHELXL97*.

## Supplementary Material

Crystal structure: contains datablock(s) I, global. DOI: 10.1107/S1600536812004138/tk5053sup1.cif

Structure factors: contains datablock(s) I. DOI: 10.1107/S1600536812004138/tk5053Isup2.hkl

Supplementary material file. DOI: 10.1107/S1600536812004138/tk5053Isup3.cml

Additional supplementary materials:  crystallographic information; 3D view; checkCIF report

## Figures and Tables

**Table 1:** Hydrogen-bond geometry (Å, °)

*D*—H⋯*A*	*D*—H	H⋯*A*	*D*⋯*A*	*D*—H⋯*A*
O1—H1⋯O22*A*^i^	0.92 (4)	2.09 (4)	2.944 (2)	155 (3)
N22—H22⋯O1*A*^ii^	0.84 (2)	1.94 (2)	2.692 (2)	150 (2)

Symmetry codes: (i) 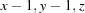; (ii) 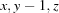.

**Fig. 1.**
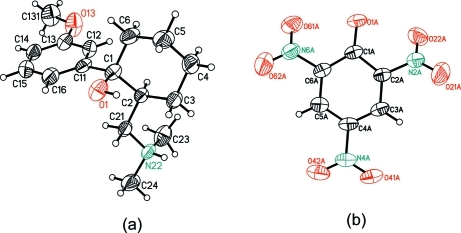
Anisotropic ellipsoid representation of the ionic components of the salt 1, together with atom labelling scheme. The ellipsoids are drawn at 50% probability level, and hydrogen atoms are depicted as spheres with arbitrary radii.

**Fig. 2.**
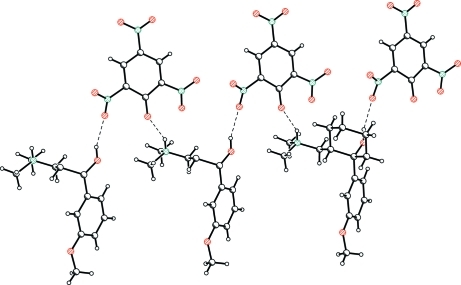
The hydrogen-bonded chain of cations and anions as seen approximately along [001]. Hydrogen bonds are drawn as dashed lines.

## supplementary crystallographic information

### Comment

Tramadol, systematic name, 2-((dimethylamino)methyl)-1-(3-methoxyphenyl) cyclohexanol, is classified as a central nervous system drug usually marketed as the hydrochloride salt. Tramadol hydrochloride is a centrally acting opioid analgesic, used in treating moderate to severe pain. The drug has a wide range of applications, including for the treatment for restless leg syndrome and fibromyalgia. Tramadol is a synthetic analog of the phenanthrene alkaloid codeine and as such, is an opioid. A review on the use of tramadol in perioperative pain is published (Scott & Perry, 2000). We have recently reported the crystal structure of tramadolium chloride - benzoic acid (1/1) (Siddaraju *et al.*, 2011), also the structure of trimadolium salicylate is known (Bica *et al.*, 2010). In view of the importance of tramadol, the paper reports the crystal structure of the tramadol picrate (1, Scheme 1), C_16_H_26_O_2_N^+^.C_6_H_2_N_3_O_7_^-^.

The protonation of tramadol molecule takes place at the *sp*^3^ nitrogen atom (the hydrogen atom was found in difference Fourier map and successfully refined), and results in the quaternary ammonium cation. The cyclohexane ring adopts the typical chair conformation (Fig. 1). The deviations from the ideal *D*_3d_ symmetry are quite small, and the values of the asymmetry parameters (Duax & Norton, 1975) are smaller than 1.7°. The hydroxy substituent is in an axial position (the C5—C6—C1—O1 torsion angle is 65.3 (3)°), while the other two are in equatorial positions [C5—C6—C1—C11 is -177.6 (2)° and C4—C3—C2—C21 is -177.70 (19)°]. The six-membered ring of the picrate anion is planar (Fig. 1) within 0.019 (1) Å. The nitro group at the position four is almost co-planar with the ring plane [dihedral angle of 2.4 (3)°], while the other two groups, due to the steric hindrance, are twisted by 37.0 (2)° and 27.3 (2)°. The C—O(-) bond length is typical for the picrate anion as the mean value for 542 organic hits from the CSD (Allen 2002, Ver. 5.33 Nov 2011) is 1.249 (16) Å.

In the crystal structure the O—H···O and N—H···O hydrogen bonds link the cations and anions into supramolecular chains along [100] (Fig. 2 and Table 1).

### Experimental

Tramadol hydrochloride (2.84 g, 0.01 mol) was dissolved in 10 ml of methanol and picric acid (1.23 g, 0.01 mol) was dissolved in 10 ml of methanol. The solutions were mixed and stirred in a beaker at 333 K for 30 minutes. The mixture was kept aside for three days at room temperature. The product formed was recrystallized from dimethyl sulphoxide by slow evaporation (M.pt.: 491–493 K).

### Refinement

Hydrogen atoms from C131 methyl groups were put in calculated positions and refined as a riding model with *U*_iso_ = 1.5 times *U*_eq_(C131). All other hydrogen atoms were located in difference Fourier maps and refined isotropically.

### Crystal data

**Table:**

C_16_H_26_NO_2_^+^·C_6_H_2_N_3_O_7_^−^	*Z* = 2
*M**_r_* = 492.48	*F*(000) = 520
Triclinic, *P*1	*D*_x_ = 1.373 Mg m^−^^3^
Hall symbol: -P 1	Mo *K*α radiation, λ = 0.7107 Å
*a* = 8.5674 (10) Å	Cell parameters from 3082 reflections
*b* = 12.3664 (12) Å	θ = 3.0–28.1°
*c* = 13.2276 (13) Å	µ = 0.11 mm^−^^1^
α = 113.003 (9)°	*T* = 295 K
β = 107.686 (10)°	Block, colourless
γ = 95.541 (9)°	0.35 × 0.2 × 0.15 mm
*V* = 1191.4 (2) Å^3^	

### Data collection

**Table:**

Agilent Xcalibur Eos diffractometer	4838 independent reflections
Radiation source: Enhance (Mo) X-ray Source	3621 reflections with *I* > 2s(*I*)
Graphite monochromator	*R*_int_ = 0.011
Detector resolution: 16.1544 pixels mm^-1^	θ_max_ = 28.2°, θ_min_ = 3.0°
ω–scan	*h* = −10→10
Absorption correction: multi-scan (*CrysAlis PRO*; Agilent, 2011)	*k* = −15→11
*T*_min_ = 0.974, *T*_max_ = 1.000	*l* = −16→17
8566 measured reflections	

### Refinement

**Table:**

Refinement on *F*^2^	Primary atom site location: structure-invariant direct methods
Least-squares matrix: full	Secondary atom site location: difference Fourier map
*R*[*F*^2^ > 2σ(*F*^2^)] = 0.052	Hydrogen site location: inferred from neighbouring sites
*wR*(*F*^2^) = 0.151	H atoms treated by a mixture of independent and constrained refinement
*S* = 1.02	*w* = 1/[σ^2^(*F*_o_^2^) + (0.0712*P*)^2^ + 0.4321*P*] where *P* = (*F*_o_^2^ + 2*F*_c_^2^)/3
4838 reflections	(Δ/σ)_max_ = 0.005
417 parameters	Δρ_max_ = 0.37 e Å^−^^3^
0 restraints	Δρ_min_ = −0.27 e Å^−^^3^

### Special details

**Table:**

Geometry. All e.s.d.'s (except the e.s.d. in the dihedral angle between two l.s. planes) are estimated using the full covariance matrix. The cell e.s.d.'s are taken into account individually in the estimation of e.s.d.'s in distances, angles and torsion angles; correlations between e.s.d.'s in cell parameters are only used when they are defined by crystal symmetry. An approximate (isotropic) treatment of cell e.s.d.'s is used for estimating e.s.d.'s involving l.s. planes.
Refinement. Refinement of *F*^2^ against ALL reflections. The weighted *R*-factor *wR* and goodness of fit *S* are based on *F*^2^, conventional *R*-factors *R* are based on *F*, with *F* set to zero for negative *F*^2^. The threshold expression of *F*^2^ > σ(*F*^2^) is used only for calculating *R*-factors(gt) *etc*. and is not relevant to the choice of reflections for refinement. *R*-factors based on *F*^2^ are statistically about twice as large as those based on *F*, and *R*- factors based on ALL data will be even larger.

### Fractional atomic coordinates and isotropic or equivalent isotropic displacement parameters (Å^2^)

**Table:**

	*x*	*y*	*z*	*U*_iso_*/*U*_eq_	
C1A	0.9734 (3)	0.89229 (17)	0.68387 (17)	0.0403 (4)	
O1A	0.9951 (2)	1.00270 (12)	0.71283 (15)	0.0588 (4)	
C2A	1.1124 (3)	0.83738 (17)	0.71022 (17)	0.0403 (4)	
N2A	1.2837 (2)	0.91273 (16)	0.76034 (16)	0.0498 (4)	
O21A	1.3924 (2)	0.8969 (2)	0.8330 (2)	0.0913 (7)	
O22A	1.3123 (2)	0.98591 (15)	0.72350 (16)	0.0697 (5)	
C3A	1.0940 (3)	0.71953 (19)	0.6920 (2)	0.0479 (5)	
H3A1	1.190 (3)	0.691 (2)	0.713 (2)	0.068 (7)*	
C4A	0.9333 (3)	0.64419 (18)	0.6403 (2)	0.0486 (5)	
N4A	0.9126 (3)	0.51879 (18)	0.6183 (2)	0.0681 (6)	
O41A	1.0393 (3)	0.48393 (19)	0.6489 (3)	0.1242 (11)	
O42A	0.7713 (3)	0.45180 (16)	0.5689 (2)	0.0864 (6)	
C5A	0.7912 (3)	0.68683 (19)	0.60864 (19)	0.0454 (5)	
H5A1	0.685 (3)	0.638 (2)	0.5742 (19)	0.048 (6)*	
C6A	0.8118 (3)	0.80560 (17)	0.62821 (17)	0.0414 (4)	
N6A	0.6574 (3)	0.84536 (17)	0.59322 (17)	0.0565 (5)	
O61A	0.6625 (2)	0.92878 (17)	0.56641 (18)	0.0814 (6)	
O62A	0.5283 (3)	0.7920 (2)	0.5906 (2)	0.0943 (7)	
C1	0.5866 (2)	0.31738 (17)	0.83219 (18)	0.0398 (4)	
O1	0.43718 (17)	0.22657 (14)	0.74367 (15)	0.0520 (4)	
H1	0.431 (4)	0.158 (3)	0.755 (3)	0.101 (11)*	
C11	0.5844 (2)	0.42921 (17)	0.80911 (17)	0.0387 (4)	
C12	0.7099 (3)	0.53512 (19)	0.8845 (2)	0.0461 (5)	
H12	0.793 (3)	0.539 (2)	0.947 (2)	0.057 (7)*	
C13	0.7053 (3)	0.64088 (18)	0.86989 (19)	0.0460 (5)	
O13	0.8299 (2)	0.74161 (15)	0.95557 (17)	0.0739 (5)	
C131	0.8202 (4)	0.8534 (2)	0.9519 (3)	0.0740 (8)	
H13A	0.8344	0.8507	0.8818	0.089*	
H13B	0.9077	0.9173	1.0205	0.089*	
H13C	0.7119	0.8681	0.9510	0.089*	
C14	0.5779 (3)	0.6393 (2)	0.77609 (19)	0.0469 (5)	
H14	0.576 (3)	0.709 (2)	0.764 (2)	0.053 (6)*	
C15	0.4555 (3)	0.5330 (2)	0.7000 (2)	0.0553 (6)	
H15	0.363 (3)	0.528 (2)	0.632 (2)	0.065 (7)*	
C16	0.4568 (3)	0.4293 (2)	0.7159 (2)	0.0507 (5)	
H16	0.370 (3)	0.353 (2)	0.661 (2)	0.061 (7)*	
C2	0.7448 (2)	0.26816 (17)	0.82194 (16)	0.0339 (4)	
H2	0.838 (3)	0.3376 (19)	0.8804 (18)	0.041 (5)*	
C21	0.7437 (2)	0.23599 (18)	0.69854 (17)	0.0390 (4)	
H21A	0.746 (2)	0.3074 (19)	0.6776 (17)	0.042 (5)*	
H21B	0.637 (3)	0.169 (2)	0.6343 (19)	0.047 (6)*	
N22	0.8910 (2)	0.18804 (15)	0.67745 (15)	0.0403 (4)	
H22	0.886 (3)	0.124 (2)	0.6850 (19)	0.050 (6)*	
C23	1.0577 (3)	0.2729 (3)	0.7626 (3)	0.0607 (6)	
H23A	1.056 (3)	0.349 (3)	0.759 (2)	0.080 (8)*	
H23B	1.146 (4)	0.233 (3)	0.731 (3)	0.092 (9)*	
H23C	1.071 (3)	0.284 (2)	0.846 (3)	0.073 (8)*	
C24	0.8724 (5)	0.1546 (3)	0.5527 (3)	0.0727 (9)	
H24A	0.876 (3)	0.227 (3)	0.536 (2)	0.079 (8)*	
H24B	0.964 (4)	0.114 (3)	0.537 (3)	0.092 (9)*	
H24C	0.755 (5)	0.097 (4)	0.500 (3)	0.129 (15)*	
C3	0.7531 (3)	0.16293 (19)	0.8552 (2)	0.0431 (5)	
H3A	0.663 (3)	0.095 (2)	0.796 (2)	0.052 (6)*	
H3B	0.856 (3)	0.1377 (18)	0.8509 (17)	0.041 (5)*	
C4	0.7468 (4)	0.1960 (3)	0.9774 (2)	0.0612 (7)	
H4A	0.743 (3)	0.129 (2)	0.992 (2)	0.066 (7)*	
H4B	0.852 (4)	0.265 (3)	1.044 (3)	0.081 (9)*	
C5	0.5934 (4)	0.2432 (2)	0.9878 (3)	0.0597 (6)	
H5A	0.599 (3)	0.270 (2)	1.071 (2)	0.066 (7)*	
H5B	0.493 (4)	0.174 (3)	0.940 (2)	0.073 (8)*	
C6	0.5841 (3)	0.3481 (2)	0.9559 (2)	0.0517 (5)	
H6A	0.678 (3)	0.417 (2)	1.014 (2)	0.050 (6)*	
H6B	0.481 (3)	0.378 (2)	0.961 (2)	0.059 (6)*	

### Atomic displacement parameters (Å^2^)

**Table:**

	*U*^11^	*U*^22^	*U*^33^	*U*^12^	*U*^13^	*U*^23^
C1A	0.0555 (12)	0.0352 (10)	0.0390 (10)	0.0203 (9)	0.0207 (9)	0.0206 (8)
O1A	0.0644 (10)	0.0318 (7)	0.0767 (11)	0.0196 (7)	0.0179 (8)	0.0252 (7)
C2A	0.0506 (11)	0.0348 (10)	0.0425 (10)	0.0151 (8)	0.0187 (9)	0.0218 (8)
N2A	0.0537 (11)	0.0411 (9)	0.0550 (10)	0.0141 (8)	0.0171 (9)	0.0236 (8)
O21A	0.0596 (11)	0.1014 (16)	0.1193 (17)	0.0151 (10)	0.0047 (11)	0.0776 (14)
O22A	0.0708 (11)	0.0561 (10)	0.0815 (12)	0.0009 (8)	0.0201 (9)	0.0393 (9)
C3A	0.0574 (13)	0.0408 (11)	0.0575 (13)	0.0232 (10)	0.0229 (11)	0.0299 (10)
C4A	0.0641 (13)	0.0336 (10)	0.0583 (13)	0.0171 (10)	0.0252 (11)	0.0273 (9)
N4A	0.0760 (15)	0.0414 (11)	0.0956 (16)	0.0172 (11)	0.0282 (13)	0.0409 (11)
O41A	0.0873 (15)	0.0650 (13)	0.233 (3)	0.0327 (12)	0.0346 (17)	0.0937 (17)
O42A	0.0896 (14)	0.0465 (10)	0.1185 (17)	0.0069 (10)	0.0238 (13)	0.0457 (11)
C5A	0.0544 (13)	0.0384 (11)	0.0448 (11)	0.0110 (10)	0.0186 (10)	0.0197 (9)
C6A	0.0509 (11)	0.0377 (10)	0.0404 (10)	0.0198 (9)	0.0180 (9)	0.0192 (8)
N6A	0.0557 (12)	0.0469 (10)	0.0601 (12)	0.0193 (9)	0.0122 (9)	0.0226 (9)
O61A	0.0756 (12)	0.0635 (11)	0.1009 (15)	0.0264 (10)	0.0071 (11)	0.0496 (11)
O62A	0.0577 (11)	0.1019 (16)	0.144 (2)	0.0319 (11)	0.0339 (12)	0.0747 (15)
C1	0.0372 (10)	0.0368 (10)	0.0487 (11)	0.0127 (8)	0.0204 (9)	0.0179 (9)
O1	0.0380 (7)	0.0442 (8)	0.0727 (10)	0.0099 (6)	0.0196 (7)	0.0256 (8)
C11	0.0431 (10)	0.0360 (9)	0.0471 (11)	0.0189 (8)	0.0276 (9)	0.0182 (8)
C12	0.0443 (11)	0.0494 (12)	0.0495 (12)	0.0198 (10)	0.0158 (10)	0.0261 (10)
C13	0.0446 (11)	0.0407 (11)	0.0565 (12)	0.0148 (9)	0.0210 (10)	0.0226 (10)
O13	0.0629 (10)	0.0461 (9)	0.0921 (13)	0.0018 (8)	0.0017 (10)	0.0336 (9)
C131	0.0787 (18)	0.0458 (13)	0.0875 (19)	0.0036 (12)	0.0190 (15)	0.0316 (13)
C14	0.0574 (13)	0.0442 (11)	0.0535 (12)	0.0249 (10)	0.0270 (11)	0.0281 (10)
C15	0.0611 (14)	0.0552 (13)	0.0488 (12)	0.0265 (11)	0.0150 (11)	0.0236 (11)
C16	0.0511 (12)	0.0459 (12)	0.0516 (12)	0.0181 (10)	0.0176 (10)	0.0178 (10)
C2	0.0339 (9)	0.0338 (9)	0.0391 (10)	0.0126 (8)	0.0170 (8)	0.0175 (8)
C21	0.0421 (10)	0.0413 (10)	0.0445 (11)	0.0213 (9)	0.0208 (9)	0.0237 (9)
N22	0.0523 (10)	0.0386 (9)	0.0508 (10)	0.0260 (8)	0.0314 (8)	0.0277 (8)
C23	0.0472 (13)	0.0650 (16)	0.090 (2)	0.0226 (12)	0.0360 (13)	0.0444 (15)
C24	0.122 (3)	0.0782 (19)	0.0646 (16)	0.062 (2)	0.0652 (19)	0.0459 (16)
C3	0.0503 (12)	0.0438 (11)	0.0523 (12)	0.0235 (10)	0.0287 (11)	0.0280 (10)
C4	0.0846 (18)	0.0710 (16)	0.0669 (16)	0.0406 (15)	0.0463 (15)	0.0500 (15)
C5	0.0804 (17)	0.0624 (15)	0.0678 (16)	0.0305 (14)	0.0517 (15)	0.0385 (13)
C6	0.0665 (15)	0.0490 (12)	0.0621 (14)	0.0274 (12)	0.0439 (13)	0.0284 (11)

### Geometric parameters (Å, º)

**Table:**

C1A—O1A	1.243 (2)	C131—H13C	0.9600
C1A—C6A	1.444 (3)	C14—C15	1.378 (3)
C1A—C2A	1.446 (3)	C14—H14	0.94 (2)
C2A—C3A	1.365 (3)	C15—C16	1.379 (3)
C2A—N2A	1.457 (3)	C15—H15	0.98 (3)
N2A—O21A	1.211 (2)	C16—H16	0.99 (2)
N2A—O22A	1.221 (2)	C2—C21	1.518 (3)
C3A—C4A	1.383 (3)	C2—C3	1.529 (3)
C3A—H3A1	0.93 (3)	C2—H2	0.97 (2)
C4A—C5A	1.386 (3)	C21—N22	1.500 (2)
C4A—N4A	1.443 (3)	C21—H21A	1.02 (2)
N4A—O42A	1.216 (3)	C21—H21B	1.04 (2)
N4A—O41A	1.220 (3)	N22—C24	1.488 (3)
C5A—C6A	1.371 (3)	N22—C23	1.490 (3)
C5A—H5A1	0.91 (2)	N22—H22	0.84 (2)
C6A—N6A	1.462 (3)	C23—H23A	0.97 (3)
N6A—O61A	1.216 (3)	C23—H23B	1.04 (3)
N6A—O62A	1.216 (3)	C23—H23C	1.03 (3)
C1—O1	1.434 (2)	C24—H24A	1.01 (3)
C1—C11	1.529 (3)	C24—H24B	1.01 (3)
C1—C6	1.536 (3)	C24—H24C	1.02 (4)
C1—C2	1.559 (2)	C3—C4	1.524 (3)
O1—H1	0.92 (4)	C3—H3A	0.96 (2)
C11—C16	1.378 (3)	C3—H3B	0.97 (2)
C11—C12	1.386 (3)	C4—C5	1.510 (3)
C12—C13	1.397 (3)	C4—H4A	0.92 (3)
C12—H12	0.89 (2)	C4—H4B	1.05 (3)
C13—O13	1.371 (3)	C5—C6	1.515 (3)
C13—C14	1.373 (3)	C5—H5A	1.01 (3)
O13—C131	1.411 (3)	C5—H5B	0.99 (3)
C131—H13A	0.9600	C6—H6A	0.97 (2)
C131—H13B	0.9600	C6—H6B	1.00 (3)
			
O1A—C1A—C6A	125.64 (18)	C11—C16—H16	117.3 (14)
O1A—C1A—C2A	122.28 (19)	C15—C16—H16	122.6 (14)
C6A—C1A—C2A	111.98 (16)	C21—C2—C3	113.46 (15)
C3A—C2A—C1A	124.3 (2)	C21—C2—C1	108.86 (15)
C3A—C2A—N2A	117.66 (18)	C3—C2—C1	111.17 (15)
C1A—C2A—N2A	118.00 (16)	C21—C2—H2	110.4 (12)
O21A—N2A—O22A	122.9 (2)	C3—C2—H2	109.8 (12)
O21A—N2A—C2A	118.80 (18)	C1—C2—H2	102.6 (12)
O22A—N2A—C2A	118.19 (17)	N22—C21—C2	114.14 (15)
C2A—C3A—C4A	119.19 (19)	N22—C21—H21A	104.9 (11)
C2A—C3A—H3A1	119.3 (16)	C2—C21—H21A	113.9 (11)
C4A—C3A—H3A1	121.5 (16)	N22—C21—H21B	105.2 (12)
C3A—C4A—C5A	121.13 (19)	C2—C21—H21B	111.3 (12)
C3A—C4A—N4A	119.63 (19)	H21A—C21—H21B	106.7 (16)
C5A—C4A—N4A	119.2 (2)	C24—N22—C23	111.6 (2)
O42A—N4A—O41A	122.6 (2)	C24—N22—C21	109.29 (18)
O42A—N4A—C4A	119.4 (2)	C23—N22—C21	113.29 (17)
O41A—N4A—C4A	118.0 (2)	C24—N22—H22	106.7 (16)
C6A—C5A—C4A	119.0 (2)	C23—N22—H22	108.3 (16)
C6A—C5A—H5A1	119.3 (14)	C21—N22—H22	107.3 (16)
C4A—C5A—H5A1	121.8 (14)	N22—C23—H23A	106.9 (17)
C5A—C6A—C1A	124.27 (18)	N22—C23—H23B	104.6 (17)
C5A—C6A—N6A	116.47 (19)	H23A—C23—H23B	112 (2)
C1A—C6A—N6A	119.22 (17)	N22—C23—H23C	108.7 (15)
O61A—N6A—O62A	122.7 (2)	H23A—C23—H23C	110 (2)
O61A—N6A—C6A	119.0 (2)	H23B—C23—H23C	114 (2)
O62A—N6A—C6A	118.2 (2)	N22—C24—H24A	111.9 (16)
O1—C1—C11	106.66 (16)	N22—C24—H24B	108.4 (18)
O1—C1—C6	109.89 (17)	H24A—C24—H24B	111 (2)
C11—C1—C6	109.94 (16)	N22—C24—H24C	107 (2)
O1—C1—C2	109.06 (15)	H24A—C24—H24C	107 (3)
C11—C1—C2	111.76 (15)	H24B—C24—H24C	111 (3)
C6—C1—C2	109.49 (16)	C4—C3—C2	112.42 (18)
C1—O1—H1	110 (2)	C4—C3—H3A	111.1 (14)
C16—C11—C12	118.14 (19)	C2—C3—H3A	108.7 (14)
C16—C11—C1	121.59 (18)	C4—C3—H3B	111.0 (12)
C12—C11—C1	120.25 (18)	C2—C3—H3B	108.2 (12)
C11—C12—C13	121.5 (2)	H3A—C3—H3B	105.2 (18)
C11—C12—H12	121.1 (16)	C5—C4—C3	111.5 (2)
C13—C12—H12	117.4 (16)	C5—C4—H4A	108.5 (16)
O13—C13—C14	124.68 (19)	C3—C4—H4A	110.2 (16)
O13—C13—C12	115.64 (19)	C5—C4—H4B	106.0 (16)
C14—C13—C12	119.6 (2)	C3—C4—H4B	111.9 (16)
C13—O13—C131	117.77 (19)	H4A—C4—H4B	109 (2)
O13—C131—H13A	109.5	C4—C5—C6	111.4 (2)
O13—C131—H13B	109.5	C4—C5—H5A	108.8 (14)
H13A—C131—H13B	109.5	C6—C5—H5A	110.1 (15)
O13—C131—H13C	109.5	C4—C5—H5B	107.8 (16)
H13A—C131—H13C	109.5	C6—C5—H5B	114.4 (16)
H13B—C131—H13C	109.5	H5A—C5—H5B	104 (2)
C13—C14—C15	118.5 (2)	C5—C6—C1	113.27 (18)
C13—C14—H14	120.5 (14)	C5—C6—H6A	109.7 (14)
C15—C14—H14	121.0 (14)	C1—C6—H6A	108.8 (13)
C14—C15—C16	122.0 (2)	C5—C6—H6B	111.5 (14)
C14—C15—H15	121.1 (15)	C1—C6—H6B	108.6 (13)
C16—C15—H15	116.8 (15)	H6A—C6—H6B	104.6 (19)
C11—C16—C15	120.1 (2)		
			
O1A—C1A—C2A—C3A	172.5 (2)	C6—C1—C11—C12	57.8 (2)
C6A—C1A—C2A—C3A	−4.0 (3)	C2—C1—C11—C12	−64.0 (2)
O1A—C1A—C2A—N2A	−7.1 (3)	C16—C11—C12—C13	2.3 (3)
C6A—C1A—C2A—N2A	176.48 (16)	C1—C11—C12—C13	−175.95 (17)
C3A—C2A—N2A—O21A	−35.3 (3)	C11—C12—C13—O13	175.22 (19)
C1A—C2A—N2A—O21A	144.2 (2)	C11—C12—C13—C14	−2.8 (3)
C3A—C2A—N2A—O22A	141.8 (2)	C14—C13—O13—C131	4.6 (3)
C1A—C2A—N2A—O22A	−38.6 (3)	C12—C13—O13—C131	−173.3 (2)
C1A—C2A—C3A—C4A	2.9 (3)	O13—C13—C14—C15	−176.5 (2)
N2A—C2A—C3A—C4A	−177.54 (18)	C12—C13—C14—C15	1.3 (3)
C2A—C3A—C4A—C5A	−1.0 (3)	C13—C14—C15—C16	0.5 (3)
C2A—C3A—C4A—N4A	178.7 (2)	C12—C11—C16—C15	−0.5 (3)
C3A—C4A—N4A—O42A	−177.3 (2)	C1—C11—C16—C15	177.77 (19)
C5A—C4A—N4A—O42A	2.3 (4)	C14—C15—C16—C11	−0.9 (3)
C3A—C4A—N4A—O41A	1.6 (4)	O1—C1—C2—C21	58.7 (2)
C5A—C4A—N4A—O41A	−178.7 (3)	C11—C1—C2—C21	−59.0 (2)
C3A—C4A—C5A—C6A	0.6 (3)	C6—C1—C2—C21	178.94 (17)
N4A—C4A—C5A—C6A	−179.1 (2)	O1—C1—C2—C3	−67.0 (2)
C4A—C5A—C6A—C1A	−2.1 (3)	C11—C1—C2—C3	175.30 (16)
C4A—C5A—C6A—N6A	−179.87 (18)	C6—C1—C2—C3	53.2 (2)
O1A—C1A—C6A—C5A	−172.8 (2)	C3—C2—C21—N22	−56.1 (2)
C2A—C1A—C6A—C5A	3.5 (3)	C1—C2—C21—N22	179.59 (16)
O1A—C1A—C6A—N6A	5.0 (3)	C2—C21—N22—C24	177.5 (2)
C2A—C1A—C6A—N6A	−178.72 (17)	C2—C21—N22—C23	−57.4 (2)
C5A—C6A—N6A—O61A	−152.9 (2)	C21—C2—C3—C4	−177.70 (19)
C1A—C6A—N6A—O61A	29.2 (3)	C1—C2—C3—C4	−54.6 (2)
C5A—C6A—N6A—O62A	26.1 (3)	C2—C3—C4—C5	54.9 (3)
C1A—C6A—N6A—O62A	−151.8 (2)	C3—C4—C5—C6	−54.4 (3)
O1—C1—C11—C16	−1.3 (2)	C4—C5—C6—C1	55.6 (3)
C6—C1—C11—C16	−120.4 (2)	O1—C1—C6—C5	65.3 (2)
C2—C1—C11—C16	117.8 (2)	C11—C1—C6—C5	−177.6 (2)
O1—C1—C11—C12	176.91 (16)	C2—C1—C6—C5	−54.5 (3)

### Hydrogen-bond geometry (Å, º)

**Table:**

*D*—H···*A*	*D*—H	H···*A*	*D*···*A*	*D*—H···*A*
O1—H1···O22*A*^i^	0.92 (4)	2.09 (4)	2.944 (2)	155 (3)
N22—H22···O1*A*^ii^	0.84 (2)	1.94 (2)	2.692 (2)	150 (2)

Symmetry codes: (i) *x*−1, *y*−1, *z*; (ii) *x*, *y*−1, *z*.
